# Discovery of catalytically active orthologues of the Parkinson's disease kinase PINK1: analysis of substrate specificity and impact of mutations

**DOI:** 10.1098/rsob.110012

**Published:** 2011-11

**Authors:** Helen I. Woodroof, Joe H. Pogson, Mike Begley, Lewis C. Cantley, Maria Deak, David G. Campbell, Daan M. F. van Aalten, Alexander J. Whitworth, Dario R. Alessi, Miratul M. K. Muqit

**Affiliations:** 1 MRC Protein Phosphorylation Unit, College of Life Sciences, University of Dundee, Dundee DD1 5EH, UK; 2 Division of Cell Signalling and Immunology, College of Life Sciences, University of Dundee, Dundee DD1 5EH, UK; 3 College of Medicine, Dentistry and Nursing, University of Dundee, Dundee DD1 5EH, UK; 4 MRC Centre for Development and Biomedical Genetics and Department of Biomedical Sciences, University of Sheffield, Sheffield S10 2TN, UK; 5 Department of Systems Biology, Harvard Medical School, Boston, MA 02115, USA

**Keywords:** biochemistry, Parkinson's disease, kinase

## Abstract

Missense mutations of the phosphatase and tensin homolog (PTEN)-induced kinase 1 (PINK1) gene cause autosomal-recessive Parkinson's disease. To date, little is known about the intrinsic catalytic properties of PINK1 since the human enzyme displays such low kinase activity *in vitro*. We have discovered that, in contrast to mammalian PINK1, insect orthologues of PINK1 we have investigated—namely *Drosophila melanogaster* (dPINK1)*, Tribolium castaneum* (TcPINK1) and *Pediculus humanus corporis* (PhcPINK1)—are active as judged by their ability to phosphorylate the generic substrate myelin basic protein. We have exploited the most active orthologue, TcPINK1, to assess its substrate specificity and elaborated a peptide substrate (PINKtide, KKWIpYRRSPRRR) that can be employed to quantify PINK1 kinase activity. Analysis of PINKtide variants reveal that PINK1 phosphorylates serine or threonine, but not tyrosine, and we show that PINK1 exhibits a preference for a proline at the +1 position relative to the phosphorylation site. We have also, for the first time, been able to investigate the effect of Parkinson's disease-associated PINK1 missense mutations, and found that nearly all those located within the kinase domain, as well as the C-terminal non-catalytic region, markedly suppress kinase activity. This emphasizes the crucial importance of PINK1 kinase activity in preventing the development of Parkinson's disease. Our findings will aid future studies aimed at understanding how the activity of PINK1 is regulated and the identification of physiological substrates.

## 2. Introduction

Mutations in the protein kinase PINK1 (PTEN-induced kinase 1) gene cause hereditary Parkinson's disease [1]. Patients harbouring PINK1 mutations typically present with early-onset Parkinson's disease, with a mean age of onset in their 30s [2]. Currently, Parkinson's disease remains incurable and treatment is aimed at relieving symptoms.

**Figure 1.**
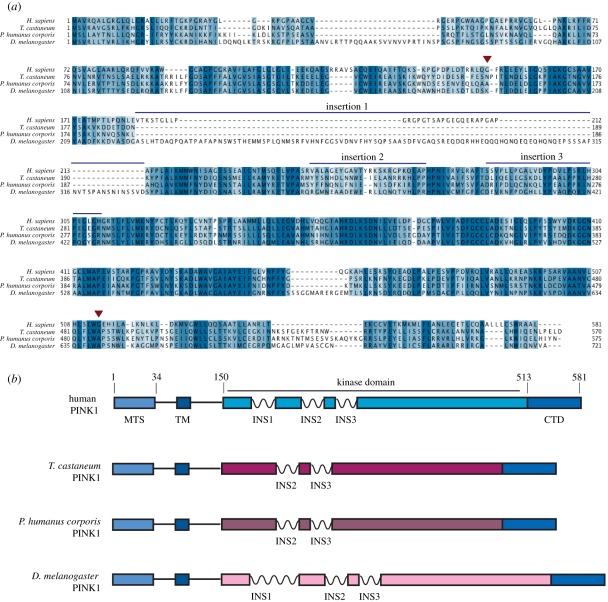
Identification of orthologues of PINK1. (*a*) Multiple sequence alignment of PINK1 from human, *T. castaneum*, *P. humanus corporis* and *D. melanogaster*. The start and the end of the kinase domain are indicated by red arrows, and the three insertions within the kinase domain discussed in the text are marked with blue bars. (*b*) Schematic of domain structure of PINK1 orthologues in human, *T. castaneum*, *P. humanus corporis* and *D. melanogaster*, with numbering corresponding to the human sequence. MTS, mitochondrial targeting sequence; TM, transmembrane helix; INS, insertion; CTD, C-terminal domain.

PINK1 encodes a protein of 581 residues, and is unique among all kinases as it is targeted to the mitochondria by an N-terminal mitochondrial targeting sequence (residues 1–34) and a transmembrane α-helix (residues 94–110) [3]. The catalytic domain of PINK1 (residues 150–513) is not closely related to other protein kinases as it features three unique insertions between the beta strands that make up the typical fold of the N-lobe of protein kinases (figure 1; electronic supplementary material, figure S1). In addition, PINK1 contains a C-terminal non-catalytic region of unknown function (residues 514–581).

The function of PINK1 remains poorly understood, although, in mammalian cells, several studies suggest that PINK1 controls another Parkinson's disease-associated enzyme, namely the parkin E3 ligase, by recruiting it to the mitochondrial membrane through an as-yet-undefined mechanism [4–7]. While PINK1 knock-out mice display no significant phenotype [8], *Drosophila* PINK1 null flies exhibit a striking phenotype sharing many overlapping behavioural and cellular features with human Parkinson's disease, including motor deficits, neuronal loss and mitochondrial abnormalities [9,10]. This phenotype is rescued by over-expression of wild-type (but not kinase-inactive) *Drosophila* PINK1 [11] and can also be rescued by over-expression of human wild-type PINK1 [12,13].

A major obstacle in studying PINK1 has been the difficulty in assessing its intrinsic catalytic properties since recombinant human PINK1 displays no significant activity. In this study, we have discovered that, in contrast to human PINK1, the three insect orthologues of PINK1 that we have examined display significant protein kinase activity when expressed in *Escherichia coli*. This has enabled us for the first time to investigate the substrate specificity of PINK1 and demonstrate that most Parkinson's disease-associated missense mutations ablate or markedly inhibit PINK1 kinase activity. These findings provide the first insights into some of the intrinsic properties of PINK1 and emphasize the crucial requirement of intact PINK1 kinase activity in preventing the development of Parkinson's disease. These findings will be helpful for future investigation of PINK1 and its role in Parkinson's disease.

## 3. Results

### 3.1. Identification of insect PINK1 orthologues that are active protein kinases

**Figure 2.**
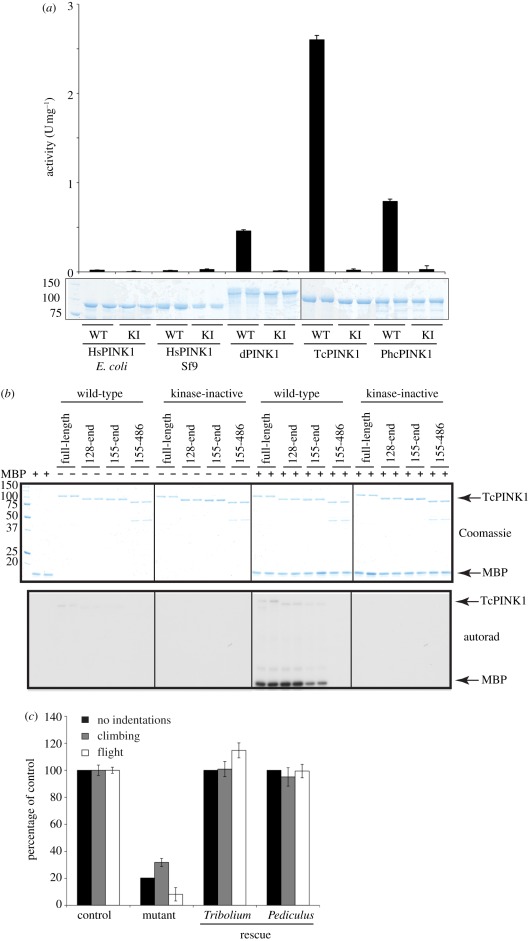
Characterization of active insect orthologues of PINK1. (*a*) Assessment of activity of wild-type N-terminally truncated human PINK1 (125–581) expressed in *E. coli* and Sf9 cells, full-length *D. melanogaster* PINK1 (dPINK1, 1–721), *T. castaneum* PINK1 (TcPINK1, 1–570) and *P. humanus corporis* PINK1 (PhcPINK1, 1–575), and corresponding kinase-inactive mutants (HsPINK1-D384A, dPINK1-D501A, TcPINK1-D359A, PhcPINK1-D357A) against myelin basic protein (MBP). The indicated enzymes (1 µg) were incubated in the presence of 5 µg MBP and [γ-^32^P] ATP for 30 min. Reactions were terminated by spotting on P81 paper, washing in phosphoric acid and quantifying phosphorylation of myelin basic protein. The results are presented as ±s.d. for a representative experiment undertaken in duplicate (upper panel). In the lower panel, representative Coomassie-stained gels showing the relative amounts of PINK1 enzyme used for each assay are shown. Fine dividing lines indicate that reactions were resolved on separate gels and grouped in the final figure. (*b*) Assessment of kinase activity of wild-type or kinase inactive (D359A) full-length (1–570), N-terminal truncation (128–570 and 155–570) and N- and C-terminal truncation mutants (155–486) of TcPINK1. The indicated forms of TcPINK1 (1 µg) were incubated in the presence (+) or absence (−) of myelin basic protein (2 µM) and [γ-^32^P] ATP for 30 min. Reactions were terminated by the addition of SDS sample buffer and separated by SDS-PAGE. Gels were analysed by Coomassie staining (upper panel) and incorporation of [γ-^32^P] ATP was detected by autoradiography (lower panel). Fine dividing lines indicate that reactions were resolved on separate gels and grouped in the final figure. (*c*) Analysis of *T. castaneum* and *P. humanus corporis* PINK1 function *in vivo*. TcPINK1 or PhcPINK1 was ectopically expressed in *Drosophila* lacking endogenous PINK1. Flight ability, climbing ability and presence of thoracic indentations were quantified. Genotypes are as follows. Control: *PINK1*^*B9*^/+, mutant: *PINK1*^*B9*^/Y; *da-GAL4*/+, mutant rescue: *PINK1*^*B9*^/Y; *da-GAL4*/+, *UAS-Tb.PINK1*^2a^/+ or *PINK1*^*B9*^/Y; *da-GAL4*/+, *UAS-Phc.PINK1*^*1*^/+. Data are presented as mean ± s.e.m.

The strongest evidence suggesting that PINK1 is an active kinase is the striking observation that the *Drosophila* PINK1 null phenotype can be rescued by wild-type but not by kinase-inactive PINK1 [11]. This inspired us to investigate whether insect orthologues of PINK1 from different species were active protein kinases. We studied *Drosophila* PINK1 (dPINK1) as well as two other insect species: *Tribolium castaneum* (TcPINK1) and *Pediculus humanus corporis* (PhcPINK1). TcPINK1 and PhcPINK1 lack the first insertion in the kinase domain, whereas dPINK1 possesses an insertion, which is considerably longer than that seen in human PINK1 (figure 1; electronic supplementary material, figures S1 and S2). We expressed the full-length forms of TcPINK1, PhcPINK1 and dPINK1 in *E. coli*, and tested their ability to phosphorylate myelin basic protein (MBP), a generic kinase substrate (figure 2*a*). Strikingly, the three insect PINK1 orthologues were active as judged by their ability to phosphorylate MBP (figure 2*a*). Consistent with the activity being mediated by PINK1 itself, a kinase-inactivating mutation targeting the magnesium-binding Asp residue within the catalytic domain of each orthologue abolished MBP phosphorylation. We observed that TcPINK1 and PhcPINK1 were more active than dPINK1, with TcPINK1 displaying the highest specific activity (figure 2*a*). In parallel experiments, we also tested whether the longest fragment of human PINK1 (125–581) that we were able to express in either *E. coli* or insect Sf9 cells (figure 2*a*) was active. Consistent with previous work, we found that human PINK1 displayed no significant kinase activity (figure 2*a*). We also observed that two reported PINK1 interactors, TRAP1 [14] and Omi [15], were not phosphorylated by insect or human PINK1 (electronic supplementary material, figure S3).

We next expressed full-length as well as truncated forms of TcPINK1 lacking the N-terminal and/or C-terminal domains in *E. coli* (figure 2*b*). Full-length or fragments lacking the non-catalytic N-terminal domain of TcPINK1 robustly phosphorylated MBP; however, deletion of the C-terminal non-catalytic domain of TcPINK1 abolished MBP phosphorylation, suggesting that this region is required for activity (figure 2*b*). Furthermore, TcPINK1 phosphorylated MBP to a greater extent than histone H1 and casein (electronic supplementary material, figure S4). Full-length PhcPINK1 also phosphorylated MBP and histone H1 similarly to TcPINK1 (electronic supplementary material, figure S4). Human PINK1, expressed in either *E. coli* or Sf9 cells, failed to phosphorylate any of these substrates (electronic supplementary material, figure S5).

We also noted that both wild-type (but not kinase-inactive) TcPINK1 and PhcPINK1 underwent autophosphorylation when incubated with Mg-ATP (figure 2*b*; see also electronic supplementary material, figures S4 and S5). We mapped the sites of autophosphorylation in wild-type TcPINK1 after incubation with Mg-ATP by LC-MS-MS on a 4000 QTRAP mass spectrometer using precursor ion scanning to detect the loss of an HPO_3_- ion (−79) from a peptide. This led to the detection of a peptide in which one of two nearby serine residues, Ser205 and Ser207, was phosphorylated, although it was not possible to unambiguously decipher which of these residues was autophosphorylated (electronic supplementary material, figure S6*a*). These Ser residues are located within the N-lobe of the kinase domain of PINK1 (figure 1*a*; electronic supplementary material, figure S1). Mutation of Ser205 to Ala led to a significant reduction in PINK1 kinase activity, whereas mutation of Ser207 only modestly reduced kinase activity (electronic supplementary material, figure S6*b*). The double Ser205Ala + Ser207Ala mutant possessed lowered activity similar to the single Ser205Ala mutant (electronic supplementary material, figure S6*b*). The Ser205 and Ser207 residues are conserved in mammalian PINK1 (Ser228 and Ser230 in human PINK1, respectively), and further work would be required to assess the importance of autophosphorylation of these residues.

### 3.2. TcPINK1 and PhcPINK1 rescue the locomotor phenotype in PINK1-deficient *Drosophila*

To verify that TcPINK1 and PhcPINK1 were true orthologues of PINK1, we tested whether TcPINK1 or PhcPINK1 could rescue locomotor defects in *Drosophila* PINK1 null flies. Multiple transgenic lines bearing TcPINK1 or PhcPINK1 under the control of the inducible GAL4/UAS system were combined into a *PINK1* mutant background, and found to rescue the flight and climbing defects as well as the appearance of thoracic indentations that are attributed to loss of PINK1 (figure 2*c*; electronic supplementary material, figure S7).

### 3.3. Analysis of PINK1 substrate specificity and elaboration of an optimal peptide substrate, PINKtide

**Figure 3.**
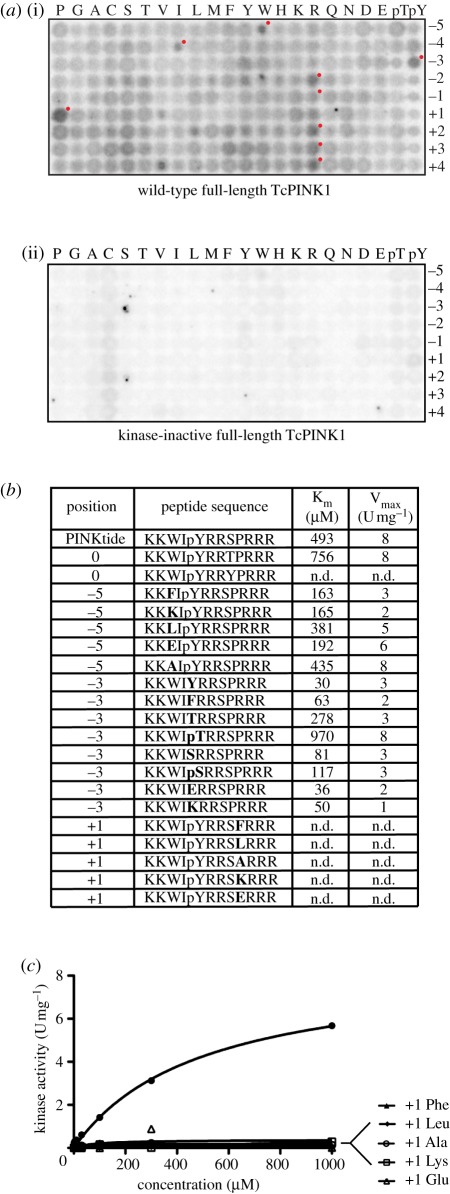
Elaboration of the PINKtide substrate for TcPINK1. (*a*) Full-length (1–570) (i) wild-type and (ii) kinase-inactive (D359A). TcPINK1 was used to screen a positional scanning peptide library of 198 biotinylated peptide libraries. Reaction products were bound to a streptavidin-coated membrane, washed and visualized by phospho-imaging. The small red dots indicate the residues selected for the PINKtide peptide sequence. (*b*) Kinetics of phosphorylation of PINKtide and indicated variants by full-length TcPINK1 (1–570). Residues that were changed relative to PINKtide are indicated in bold. *K*_m_ and *V*_max_ values were derived by nonlinear regression analysis as described in §5. n.d. denotes that a particular peptide was phosphorylated poorly and kinetic values were not determinable. Similar results were obtained in at least two experiments. (*c*) Kinetics of phosphorylation of mutants of PINKtide at the +1 position by full-length TcPINK1 (1–570) in a representative experiment to illustrate the marked preference for a +1 Pro residue.

To investigate the substrate specificity of TcPINK1, we used a positional scanning library approach [16,17], in which the ability of TcPINK1 to phosphorylate 198 biotinylated peptide libraries simultaneously was assessed. Each library contains a 1 : 1 serine and threonine at the central position, and one additional position fixed to one of the 20 amino acids and phospho-threonine and phospho-tyrosine. Phospho-tyrosine and phospho-threonine were included to allow for identification of kinases requiring priming phosphorylation events. All other positions contain an equimolar degenerate mixture of natural amino acids (except serine, threonine and cysteine). Recombinant full-length wild-type or kinase-inactive TcPINK1 were used to phosphorylate all peptide libraries in solution using [γ-^32^P] ATP, and biotinylated peptides were captured on a streptavidin-coated membrane. The relative preference for each amino acid at each position was determined by quantifying ^32^P incorporation by phospho-imaging. This revealed a preference for a proline at the +1 position and a general preference at several residues for basic residues such as arginine. There were also moderate preferences for a phospho-tyrosine at the −3 position and a tryptophan at the −5 position (figure 3*a*). In a parallel experiment, there was negligible phosphorylation of peptides for the experiments undertaken with the kinase-inactive TcPINK1 (figure 3*a*).

The data from the positional scanning peptide library suggested that the optimal TcPINK1 phosphorylation motif between the −5 and the +4 position was WIpYRR***S***PRRR, in which the underlined Ser comprises the site of phosphorylation. We observed that wild-type but not catalytically inactive TcPINK1 significantly phosphorylated this peptide (in which two Lys residues were added to the N-terminus to improve solubility), with a Km of approximately 500 µM and a *V*_max_ of approximately 10 U mg^−1^ for TcPINK1 (figure 3*b* and electronic supplementary material, figure S8). This peptide (KKWIpYRRSPRRR) was termed PINKtide. Changing the phosphorylated Ser residue to Thr did not affect phosphorylation (figure 3*b*), suggesting that PINK1 could phosphorylate substrates at either Ser or Thr residues. Mutation of the phosphorylation site to Tyr abolished phosphorylation (figure 3*b*), confirming that PINK1 is a Ser/Thr-specific kinase. TcPINK1 also failed to significantly phosphorylate a panel of 11 previously described peptide substrates used to assess catalytic activity of diverse kinases, including CDKtide, which contains a +1 proline required for phosphorylation by the CDK kinases (electronic supplementary material, figure S9).

Mutation of the Pro residue at the +1 position of PINKtide to a Phe, Leu, Ala, Lys or Glu virtually abolished phosphorylation by TcPINK1, indicating that PINK1 may possess a marked preference for phosphorylation of Ser/Thr residues that have a +1 Pro residue (figure 3*c*). We next investigated the effect of mutating the −3 and −5 residues that are the critical determinants of substrate specificity for many kinases [18]. Mutation of the −5 Trp residue to five other residues, although lowering the *K*_m_ up to threefold, concomitantly decreased *V*_max_ up to fourfold (with the exception of alanine), thus not altering steady-state kinetics significantly (figure 3*b*). Mutation of the −3 phospho-tyrosine residue to tyrosine markedly reduced the *K*_m_ (16-fold), but also lowered the *V*_max_ threefold (figure 3*b*). We also mutated the −3 residues to seven other residues but found that although many of these lowered the *K*_m_, they also resulted in significant decreases in *V*_max_ (figure 3*b*). Out of the approximately 20 peptides tested, none displayed a higher *V*_max_ value than PINKtide (figure 3*b*).

### 3.4. Impact of Parkinson's disease mutations on TcPINK1 catalytic activity

**Figure 4.**
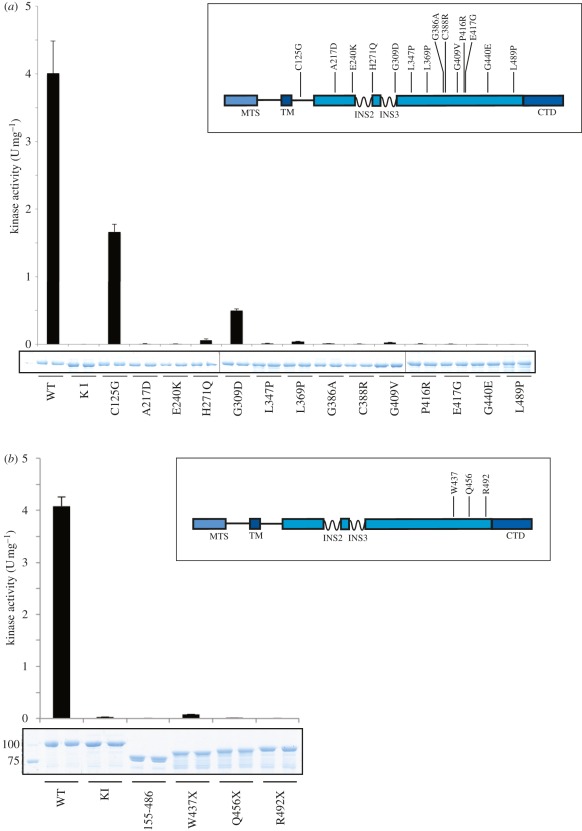
Effect of Parkinson's disease mutation on PINK1 kinase activity. (*a*) Inset: Schematic of the location of missense PINK1 mutations where the wild-type residue is conserved in both human PINK1 and TcPINK1. Numbering is according to human PINK1. Mutations were introduced into full-length TcPINK1 (1–570), and enzymes (1 µg) were incubated in presence of PINKtide (1 mM) and [γ-^32^P] ATP for 30 min. Reactions were terminated by spotting onto P81 paper, washing in phosphoric acid and quantifying phosphorylation of PINKtide bound to P81 paper. The results are presented as ±s.d. for three experiments undertaken in duplicate. Representative Coomassie-stained gels showing the relative amounts of PINK1 enzyme used for each assay are shown. (*b*) Inset: Schematic of the location of C-terminally truncating PINK1 mutations. Numbering is according to human PINK1. Mutations were introduced into full-length TcPINK1 (1–570), enzymes (1 µg) were incubated in presence of PINKtide (1 mM) and [γ-^32^P] ATP for 30 min. Reactions were terminated by spotting onto P81 paper, washing in phosphoric acid and quantifying phosphorylation of PINKtide bound to P81 paper. The results are presented as ±s.d. for two experiments undertaken in duplicate. Representative Coomassie-stained gels showing the relative amounts of PINK1 enzyme used for each assay are shown.

We determined the effects of Parkinson's disease-associated mutations on PINK1 catalytic activity by selecting 14 homozygous or compound heterozygous missense mutations that lead to early-onset Parkinson's disease in which the disease-associated amino acid residue is conserved in TcPINK1 (figure 4*a*; electronic supplementary material, table S1). All bar one of the selected mutations are located within the kinase domain (figure 4*a*, inset). We observed that all mutants investigated were expressed in *E. coli* at comparable levels and purity to wild-type TcPINK1 (figure 4*a*). Measuring specific activity against both PINKtide (figure 4*a*) and MBP (electronic supplementary material, figure S10) revealed that 12 mutations lying within the kinase domain (A217D, E240K, H271Q, L347P, L369P, G386A, C388R, G409V, P416R, E417G, G440E and L489P) virtually abolished or ablated kinase activity. One mutation, G309D, which lies within the third kinase domain insertion, reduced kinase activity by 90 per cent. The only other mutation studied that was not situated within the kinase domain (C125G) only moderately reduced kinase activity.

Nonsense mutations of PINK1 that lead to truncation of PINK1 and the loss of the C-terminal non-catalytic domain, as well as a small portion of the kinase domain, have also been reported (figure 4*b*, inset). Two of these mutations, W437X (originally found in the Marsala kindred) [1] and Q456X [19], are inherited in a homozygous recessive manner, while a third, R492X, has been found as a compound heterozygous mutation [19] (electronic supplementary material, table S1). We introduced these mutations into TcPINK1 and tested their activity against either PINKtide (figure 4*b*) or MBP (electronic supplementary material, figure S11), and found that all of the truncating mutations tested abolished kinase activity.

## 4. Discussion

In contrast to human PINK1, we report that insect orthologues of PINK1, including *Drosophila melanogaster, T. castaneum* (TcPINK1) and *P. humanus corporis* (PhcPINK1), exhibit significant catalytic kinase activity *in vitro* (figure 1). Using the most active orthologue, TcPINK1, we have been able to describe the first investigation of the substrate specificity of PINK1 and we have used this information to elaborate a novel peptide substrate, KKWIpYRRSPRRR (figure 3), which we have designated PINKtide. We have employed PINKtide to probe the effect that Parkinson's disease-associated mutations exert on PINK1 kinase activity (figure 4; electronic supplementary material, figure S10). A key question concerns why PINK1 from insect species is active, whereas human PINK1 is inactive when expressed in a similar manner (figure 2*a*; electronic supplementary material, figure S5). It is possible that mammalian PINK1 is not active as it is regulated in a more complex manner, and that additional interactors and/or covalent modifications not present in *E. coli* or insect hosts (used to express human PINK1 in our studies) are required to activate human PINK1.

Our results suggest that missense PINK1 mutations situated within the kinase domain exert their Parkinson's disease-causing effects by markedly suppressing kinase activity (figure 4*a*). This emphasizes the importance of identifying the key physiological substrates of PINK1 in order to understand how the loss of kinase activity leads to neurodegeneration in Parkinson's disease. To date, no robust substrates of PINK1 have been discovered, partly owing to the unavailability of active recombinant PINK1. We have also evaluated some of the previously described PINK1 interactors (TRAP1 [14] and Omi [15]), and found that neither of them were significantly phosphorylated *in vitro* by insect PINK1 (electronic supplementary material, figure S3), therefore suggesting that these are not directly phosphorylated by PINK1. It would also be interesting to establish whether sites of PINK1 phosphorylation identified in substrates in the future lie within Ser/Thr-Pro motifs that might be predicted from our initial analysis of PINK1 substrate specificity. We have also attempted to map the sites on myelin basic protein that were phosphorylated by TcPINK1 and found numerous sites were phosphorylated at a very low stoichiometry (data not shown). This confirms that myelin basic protein is not an optimal substrate for PINK1. We would predict that PINK1 would phosphorylate a genuine substrate at a distinct site(s) at significant stoichiometry.

The C-terminus of PINK1 has no homology to any known protein. We have found that removal of the C-terminus of TcPINK1 (486–570), equivalent to residues 513–581 in human PINK1, abolishes TcPINK1 kinase activity, and also that the three C-terminal truncating disease mutants W437X, Q456X and R492X ablate kinase activity (figure 4*b*; electronic supplementary material, figure S10). Our data are also consistent with recent *Drosophila* data showing that human wild-type PINK1 but not C-terminal-truncated human PINK1 (residues 1–509) could rescue the *Drosophila* PINK1 null phenotype [13]. It would be interesting to undertake further analysis to establish how the C-terminal domain might regulate kinase activity.

In the future, it will also be vital to solve the atomic structure of PINK1 to fully understand how the enzyme is regulated and to understand the molecular mechanism by which mutations inhibit kinase activity. The observation that the C125G mutation lying outside the kinase domain only modestly impaired kinase activity suggests that this mutation affects PINK1 function by a distinct mechanism. As the C125G mutation is close to the transmembrane mitochondrial-binding region, it could impact on recruitment of PINK1 to the mitochondrial membrane. It could also affect the interaction of PINK1 with an upstream activator or physiological substrate.

Many kinase inhibitors are being developed for the treatment of diseases involving aberrant protein phosphorylation. As it has not previously been possible to assay PINK1 kinase activity, it is not known whether the numerous kinase inhibitors currently in preclinical development or clinical use may also inhibit PINK1. It should also be noted that a recent study has advocated that PINK1 inhibitors might have utility in treating certain forms of colorectal cancer with mutations in mismatch repair genes MSH2, MLH1 and MSH6 [20]. A key issue with this is whether administration of PINK1 inhibitors to human cancer patients could have the potential to induce Parkinson's disease. In the literature, the youngest age of the onset of Parkinson's disease in patients harbouring homozygous PINK1 mutations is approximately 10 (A217D mutation) [21], suggesting that at least a decade of PINK1 inhibition is required before Parkinson's disease symptoms develop. It is thus likely that short periods of exposure to compounds that inhibit PINK1 for cancer treatments would carry a reduced risk of Parkinson's disease compared with chronic long-term treatment. Furthermore, chemical inhibition of kinases rarely achieves a null effect and more often confers a hypomorphic effect on kinase activity. Taking these factors into consideration, we feel that PINK1 inhibitors would be associated with a lower risk compared with disease-causing Parkinson's disease mutations that inactivate PINK1 (figure 4*a*; electronic supplementary material, figure S10). We also suggest that insect PINK1 could be introduced into kinase panels used to profile kinase inhibitors being developed for the treatment of human disease, at least until it is worked out how to activate and assay human PINK1. Insect PINK1 could also be deployed to attempt to identify PINK1 inhibitors for the treatment of cancer, although further work would be needed to ascertain whether an inhibitor of insect PINK1 would also effectively suppress mammalian PINK1.

In conclusion, we report for the first time a novel method to express an active form of PINK1. This has enabled us to develop an assay to quantitatively assess PINK1 activity and investigate its substrate specificity. Our work suggests that, with the exception of the C125G mutation, all other Parkinson's disease mutations assessed are likely to exert their disease-causing effects by suppressing kinase catalytic activity. These observations emphasize the importance of PINK1 kinase activity in preventing the onset of Parkinson's disease, and that the key challenge in future will be to identify PINK1 substrates and study the relevance of these in Parkinson's disease. We hope that the results presented in this study will aid with assaying PINK1 catalytic activity and in the hunt for substrates of this enzyme.

## 5. Material and methods

### 5.1. Reagents

Amylose resin was from New England Biolabs (Ipwich, MA). [γ-^32^P] ATP was from PerkinElmer (Waltham, MA). P81 paper was from Whatman (Maidstone, UK). Bovine myelin basic protein and bovine casein were from Sigma (St Louis, MO), bovine histone H1 was from Abcam (Cambridge, UK). PINKtide and its derivatives were synthesized by GL Biochem (Shanghai, China). Restriction enzyme digests, DNA ligations and other recombinant DNA procedures were performed using standard protocols. All mutagenesis was carried out using the QuikChange site-directed-mutagenesis method (Stratagene, Santa Clara, CA) with KOD polymerase (Novagen, Merck, Whitehouse Station, NJ). All DNA constructs were verified by DNA sequencing, which was performed by The Sequencing Service, School of Life Sciences, University of Dundee, using DYEnamic ET terminator chemistry (Amersham Biosciences, GE Healthcare, Little Chalfont, UK) on Applied Biosystems (Foster City, CA) automated DNA sequencers. cDNA for *T. castaneum* PINK1 and *P. humanus corporis* PINK1 were synthesized by GenScript USA (Piscataway, NJ).

### 5.2. Buffers

Lysis buffer contained 50 mM Tris–HCl (pH 7.5), 150 mM NaCl, 1 mM EDTA, 1 mM ethylene glycol tetraacetic acid (EGTA), 5 per cent (v/v) glycerol, 1 per cent (v/v) Triton X-100, 0.1 per cent (v/v) 2-mercaptoethanol, 1 mM benzamidine and 0.1 mM phenylmethylsulphonyl fluoride (PMSF). Wash buffer contained 50 mM Tris–HCl (pH 7.5), 500 mM NaCl, 0.1 mM EGTA, 5 per cent (v/v) glycerol, 0.03 per cent (v/v) Brij-35, 0.1 per cent (v/v) 2-mercaptoethanol, 1 mM benzamidine and 0.1 mM PMSF. Equilibration buffer contained 50 mM Tris–HCl (pH 7.5), 150 mM NaCl, 0.1 mM EGTA, 5 per cent (v/v) glycerol, 0.03 per cent (v/v) Brij-35, 0.1 per cent (v/v) 2-mercaptoethanol, 1 mM benzamidine and 0.1 mM PMSF. Elution buffer was equilibration buffer with the addition of 12 mM maltose. Storage buffer was equilibration buffer with the addition of 0.27 M sucrose, and glycerol, PMSF and benzamidine were omitted.

### 5.3. Expression and purification of recombinant kinases

All PINK1 enzymes used in this study were expressed in *E. coli* as maltose-binding protein fusion (MaBP) proteins. In addition, human MaBP-PINK1 (125-end) was also expressed in Sf9 cells. Briefly, BL21 Codon+ transformants were grown at 37°C to an OD_600_ of 0.3, shifted to 16°C and induced with 250 µM IPTG (isopropyl β-d-thiogalactoside) at OD_600_ = 0.5. Cultures were then grown for a further 15–16 h at 16°C. Cells were lysed by sonication in lysis buffer. Lysates were clarified by centrifugation at 30 000*g* for 30 min at 4°C followed by incubation with 1 ml per litre of culture of amylose resin for 1.5 h at 4°C. The resin was washed thoroughly in wash buffer, then equilibration buffer, and proteins were then eluted. Proteins were dialysed overnight at 4°C into storage buffer, snap frozen and stored at −80°C until use. Expression of human PINK1 in Sf9 cells was conducted as follows. Sf9 cells were seeded at a density of 1.5 × 10^6^ cells ml^−1^ and 1 : 10 P2 virus was added. Cells were incubated in spinner flasks in the dark at 27°C for 4 days, after which point the cells were harvested and the protein was purified using the MaBP affinity tag as above. Bacmids were produced and viruses amplified according to standard protocols.

### 5.4. Kinase assays

Assays using protein substrates were set up in a volume of 40 µl, with substrates at 2 µM and all kinases at 1 µg in 50 mM Tris–HCl (pH 7.5), 0.1 mM EGTA, 10 mM MgCl_2_, 2 mM dithiothreitol and 0.1 mM [γ-^32^P] ATP. Assays were incubated at 30°C with shaking at 1200 r.p.m. and terminated after 30 min by addition of sodium dodecyl sulphate (SDS) sample buffer. Reaction mixtures were resolved by sodium dodecyl sulphate polyacrylamide gel electrophoresis (SDS-PAGE). Proteins were detected by Coomassie staining. Incorporation of [γ^−32^P] ATP into substrates was analysed by autoradiography.

For assays using peptide substrates, assays were set up in a volume of 50 µl, with all kinases at 1 µM and peptide substrates at the indicated concentrations in 50 mM Tris–HCl (pH 7.5), 0.1 mM EGTA, 10 mM MgCl_2_, 2 mM dithiothreitol and 0.1 mM [γ-^32^P] ATP. Assays were incubated for 30 min at 30°C with shaking at 1200 r.p.m., and terminated by spotting of 40 µl of the reaction mixture onto P81 phosphocellulose paper and washing in 50 mM phosphoric acid. After extensive further washing, incorporation of [γ-^32^P] ATP into substrates was quantified by Cerenkov counting. For MBP assays using P81 paper, 5 µg of MBP was used.

One unit of TcPINK1 activity was defined as the amount of enzyme that catalysed the incorporation of 1 nmol of ATP into a given substrate. *K*_m_ and *V*_max_ values were determined by analysing the phosphorylation of PINKtide and its variants at various concentrations. *K*_m_ and *V*_max_ values were calculated using the GraphPad Prism program.

### 5.5. Multiple sequence alignment

PINK1 sequences were aligned with MUSCLE (MUltiple Sequence Comparison by Log-Expectation), EBI (www.ebi.ac.uk/Tools/muscle/index.html) and managed with JalView (www.jalview.org/).

### 5.6. Analysis of *Drosophila* phenotypic rescue

*Drosophila* were raised under standard conditions at 25°C on agar, cornmeal and yeast food. *PINK1*^*B9*^ mutants, *da*-GAL4 line and assays for flight, climbing and thoracic indentations have been described previously [22]. Full-length coding sequence for *Tribolium* or *Pediculus* PINK1 was cloned into the pUAST transgenesis vector and transgenic lines were generated by standard techniques in a *w*^*1118*^ background (BestGene Inc., Chino Hills, CA). Multiple independent lines were assessed for consistency.

### 5.7. Mass spectrometry

Samples were analysed by an Applied Biosystems 4000 Q-TRAP system with precursor ion scanning as described previously [23].
